# A significant increase in the pepsinogen I/II ratio is a reliable biomarker for successful *Helicobacter pylori* eradication

**DOI:** 10.1371/journal.pone.0183980

**Published:** 2017-08-30

**Authors:** Hiroki Osumi, Junko Fujisaki, Takanori Suganuma, Yusuke Horiuchi, Masami Omae, Toshiyuki Yoshio, Akiyoshi Ishiyama, Tomohiro Tsuchida, Kazumasa Miki

**Affiliations:** 1 Department of Gastroenterology, Cancer Institute Hospital, Japanese Foundation for Cancer Research, Tokyo, Japan; 2 Department of Gastroenterology, Iida Municipal Hospital, Nagano, Japan; National Cancer Center, JAPAN

## Abstract

**Background:**

*Helicobacter pylori* (*H*. *pylori)* eradication is usually assessed using the ^13^C-urea breath test (UBT), anti-*H*. *pylori* antibody and the *H*. *pylori* stool antigen test. However, a few reports have used pepsinogen (PG), in particular, the percentage change in the PG I/II ratio. Here, we evaluated the usefulness of the percentage changes in serum PG I/II ratios for determining the success of eradication therapy for *H*. *pylori*.

**Materials and methods:**

In total, 650 patients received eradication therapy from October 2008 to March 2013 in our Cancer Institute Hospital. We evaluated the relationship between *H*. *pylori* eradication and percentage changes in serum PG I/II ratios before and 3 months after treatment with CLEIA^®^ (FUJIREBIO Inc, Tokyo, Japan). The gold standard of *H*. *pylori* eradication was defined as negative by the UBT performed 3 months after completion of eradication treatment. Cut-off values for percentage changes in serum PG I/II ratios were set as +40, +25 and +10% when the serum PG I/II ratio before treatment was below 3.0, above 3.0 but below 5.0 and 5.0 or above, respectively.

**Results:**

Serum PG I and PG II levels were measured in 562 patients with *H*. *pylori* infection before and after eradication therapy. Eradication of *H*. *pylori* was achieved in 433 patients studied (77.0%). The ratios of first, second, third-line and penicillin allergy eradication treatment were 73.8% (317/429), 88.3% (99/112), 75% (12/16) and 100% (5/5), respectively. An increasing percentage in the serum levels of the PG I/II ratios after treatment compared with the values before treatment clearly distinguished success from failure of eradication (108.2±57.2 vs. 6.8±30.7, *p*<0.05). Using the above cut-off values, the sensitivity, specificity and validity for determination of *H*. *pylori* were 93.1, 93.8 and 93.2%, respectively.

**Conclusion:**

In conclusion, the percentage changes in serum PG I/II ratios are useful as evaluation criteria for assessing the success of eradication therapy for *H*. *pylori*.

## Introduction

Pepsinogen (PG) is the inactive precursor of pepsin, specifically produced in the stomach, of which 99% is secreted into the gastric lumen and 1% into the bloodstream [1] [2]. PG mainly comprises two biochemically and immunologically different isozymes (PG I and PG II). PG I is secreted only from the oxyntic mucosa, whereas PG II is secreted from the fundic pyloric and proximal duodenal glands [1] [2]. Recent prospective cohort studies confirmed measurement of serum PG levels before eradication of *Helicobacter pylori* is useful for assessing the risk of gastric cancer [3]. The production of PG I is considerably reduced or abolished in the case of atrophy of the corpus mucosa and the loss of chief cells as well as parietal cells. By contrast, the serum levels of PG II increase when the gastric mucosa is infiltrated by neutrophils and mononuclear cells in the antrum as a result of *H*. *pylori* infection and its extension into the upper stomach [1] [2]. Thus, the ratio of PG I to II decreases further in association with low PG I levels by advanced atrophic gastritis in the corpus.

In Japan, diagnosis methods of *H*. *pylori* infection are based on an invasive examination method using an endoscope (rapid urease test (RUT), microscopic examination, culturing method) and a noninvasive examination method without endoscope (^13^C-urea breath test (UBT), anti-*H*. *pylori* antibody measurement, *H*. *pylori* stool antigen test) according to the recommendation of the Japanese Society for Helicobacter Research [4–9]. If we could diagnose eradication of *H*. *pylori* infection by measuring the PG I/II ratio, it would be both cost effective and convenient. The aim of this study was to evaluate the usefulness as evaluation criteria of using the percentage changes in serum PG I/II ratios to determine the success of eradication therapy for *H*. *pylori*.

## Patients and methods

This study was performed in accordance with the Declaration of Helsinki. The Cancer Institute Hospital of Japanese Foundation for Cancer Research, Institutional Review Board approved the study (registry number: 2016–1168). Need for consent was waived by the Cancer Institute Hospital of Japanese Foundation for Cancer Research, Institutional Review Board because this research was using only clinical information without invasion or intervention of patients. Instead, we were doing information disclosure (opt-out) on the homepage in our hospital. A total of 650 patients received eradication therapy from October 2008 to March 2013 and serum PG I and PG II levels were measured in 562 cases with *H*. *pylori* infection before and after eradication therapy. The inclusion criteria was absence of known allergies for antibiotics used in either regimen. The exclusion criteria were: (1) patients treated with proton-pump inhibitor (PPI), H2 receptor antagonist or antibiotics in the 4 to 6 weeks previous to UBT (2) patients with post gastrectomy.

### Assessment of H. pylori infection and eradication therapy and assessment

*H*. *pylori* infection was confirmed by histology (two biopsies taken from the gastric antrum and from the body for examination with Hematoxylin-Eosin, Genta, and Giemsa stains), UBT (UBIT 100 mg tablets, Otsuka Pharmaceutical Co., Ltd., using a cut-off of 2.5‰), and *H*. *pylori* anti-body test (antibody determination kit, E-Plate Eiken *H*. *pylori* antibody, using a cut-off of 10 U/ml). All participants received standard of care treatment except for the third line therapy. Standard first-line triple therapy in Japan includes PPI (20 mg rabeprazole, 20 mg esomeprazole or 30 mg lansoprazole), 750 mg amoxicillin (AMPC) and 400 mg clarithromycin (CAM) twice a day for a week. Standard second-line triple therapy includes PPI (20 mg rabeprazole, 20 mg esomeprazole or 30 mg lansoprazole), 250 mg metronidazole (MNZ) and 750 mg AMPC twice a day for a week [10]. In Japan, although third-line therapy has not been established, we selected rescue regimens such as sitafloxacin (STFX)-based triple therapies (lansoprazole 30 mg bid + AMPC 750 mg bid + STFX 100 mg bid for one week, n = 1) [11], high dose PPI and AMPC-based dual therapies (rabeprazole 10 mg q.i.d. and amoxicillin 500 mg q.i.d. for 2 weeks, n = 1) [12] and quadruple therapy with ecabet sodium, omeprazole, amoxicillin and metronidazole (1 g ecabet sodium, 500 mg AMPC, four times per day, and 20 mg rabeprazole, 250 mg MNZ, twice per day, for two weeks, n = 14) [13] at the physician's choice. The Japanese guidelines for *H*. *pylori* treatment 2016 proposed penicillin allergy regimens, such as PPI/CAM/MNZ [14], PPI/STFX/MNZ [15] and PPI/MINO/MNZ [16], and we used 20 mg rabeprazole, 250 mg MNZ and 100 mg MINO twice per day for a week [13]. Assessment of *H*. *pylori* eradication was performed by the UBT at least 12 weeks after the completion of eradication therapy and the cut-off value for the UBT test was 2.5‰ [10]. Completion was defined as a patient finishing taking all medicines prescribed as part of a particular therapy. We decided to allow these medicines until 2 weeks before judgment of *H*.*pylori* eradication in this study.

### Measurement of PG I and PG II

We also determined the percentage changes in serum PG I/II ratios before and 3 months after treatment with CLEIA^®^ (FUJIREBIO Inc, Tokyo, Japan) and established cut-off values to distinguish success from failure of *H*. *pylori* eradication. Cut-off values for percentage changes in serum PG I/II ratios were set as +40, +25 and +10% when the serum PG I/II ratio before treatment was below 3.0, above 3.0 but below 5.0 and 5.0 or above, respectively [17]. The percentage change in values was calculated as follows: percentage change = {(value 3 months after the end of treatment)–(value before treatment)}/(value before treatment)×100. The gold standard of *H*. *pylori* eradication was defined as negative by the use of a UBT performed 3 months after completion of the eradication treatment.

### Statistical analysis

The statistical significance of serum levels of PG I, PG II and PG I/II ratios as a function of the eradication status were determined by the Student’s *t* test. The significance level was defined as *p*<0.05. All statistical analyses were performed using EZR (Saitama Medical Center, Jichi Medical University), a graphical user interface for R (The R Foundation for Statistical Computing, Vienna, Austria).

## Results

### Patients’ characteristics (Table 1)

**Table 1:** Patient characteristics.

	n = 562
Age (median, range)	62(26–83)
Gender (male) n,%	219(38.9)
Treatment (1^st^ /2^nd^ /3^rd^ /other)	429/112/16/5
Eradication rate n,%	433(77)
Completion rate n,%	559(99.4)
Adverse event n,%	8(1.5)

**Fig 1:**
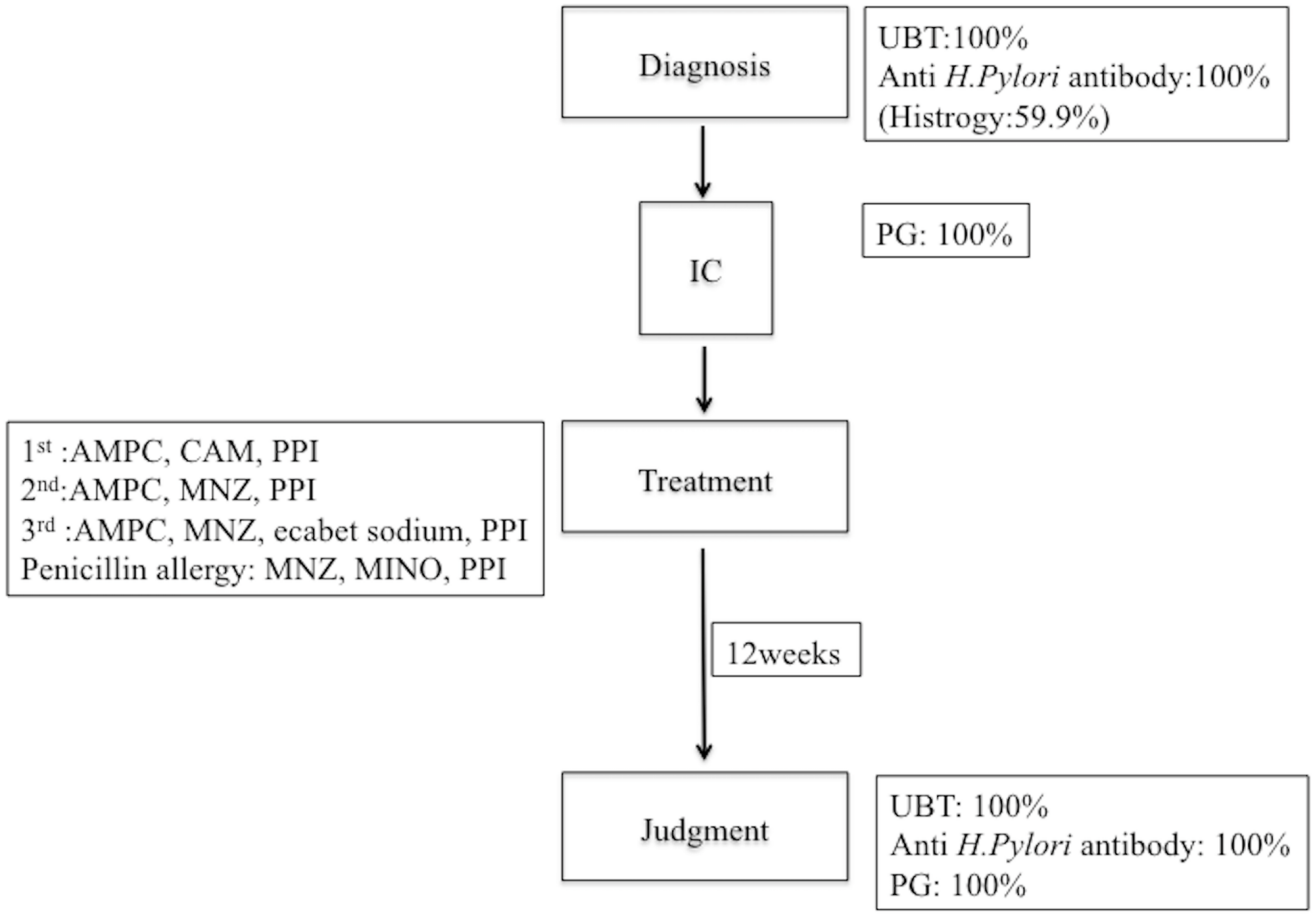
Flow chart of this study.

In total, 562 patients (336 women, 59.7%; 226 men, 40.3%; median age: 62 years, range: 26–83) were included in the study. The ratios for first, second, third-line and penicillin allergy eradication treatment were 76.3% (429/562), 19.9% (112/562), 2.8% (16/562) and 0.8% (5/562), respectively. All patients performed UBT and anti *H*.*pylori* antibody for diagnosis of *H*. *pylori* infection and judgment of *H*. *pylori* eradication and 59.9% patients performed histology for diagnosis of *H*. *pylori infection*. On the basis of the serum PG test proposed by Miki et al, when positive serum PG was PG I levels of ≤70 ng/mL and PG I/II ratios of ≤3.0, 93.5% (317/339) patients had atrophic gastritis. The consort diagram of the study is shown in Fig 1.

### Eradication results (Table 1)

Eradication of *H*. *pylori* was achieved in 433 cases (77.0%, 95% confidence interval (CI): 73.5–80.5). The eradication rates with first, second and third-line and penicillin allergy therapies were 73.8% (317/429), 95% CI: (69.6–78); 88.3% (99/112), 95% CI: (82.4–94.3); 75% (12/16), 95% CI: (53.2–96.2); 100% (5/5), respectively. The completion rate in this study was (99.4%, 95% CI: 98.8–100). Adverse events occurred in eight patients (1.5%), with skin erythema as the most common symptom (six patients).

### Serum levels of PG I, PG II and the PG I/II ratio before and 3 months after the end of treatment (Table 2)

**Table 2:** Serum levels of PG I, PG II and the PG I/II ratio before and 3 months after the end of the treatment.

	Treatment success (n = 433)	Treatment failure (n = 129)	*p*.value
PG I (pre-eradication, ng/dl)	55.0±30.6	57.9±30.2	*n.s
PG I (post-eradication, ng/dl)	33.6±19.6	54.2±26.7	<0.05
PG II (pre-eradication, ng/dl)	24.7±12.1	24.9±12.8	*n.s
PG II (post-eradication, ng/dl)	7.5±3.5	23.3±12.2	<0.05
PG I/II ratio (pre-eradication)	2.3±1.0	2.4±0.9	*n.s
PG I/II ratio (post-eradication)	4.5±1.6	2.5±1.1	<0.05
The reduction rate of PG I (%)	32.7±24.8	0.5±25.8	<0.05
The reduction rate of PG II (%)	64.9±17.4	6.7±30.7	<0.05

*n.s: not significant

There were no significant differences in the serum levels of PG I, PG II and the PG I/II ratios before eradication treatment between the eradication success group and the failure group (PG I: 55.0±30.6 ng/dl vs. 57.9±30.2 ng/dl, PG II: 24.7±12.1 ng/dl vs. 24.9±12.8 ng/dl, PG I/II: 2.3±1.0 vs. 2.4±0.9, *p* = n.s). By contrast, significant differences were detected for each factor after eradication treatment (PG I: 33.6±19.6 ng/dl vs. 54.2±26.7 ng/dl, PG II: 7.5±3.5 ng/dl vs. 23.3±12.2 ng/dl, PG I/II: 4.5±1.6 vs. 2.5±1.1, *p*<0.05).

### Percentage changes in serum levels of the PG I/II ratios (Fig 2)

**Fig 2:**
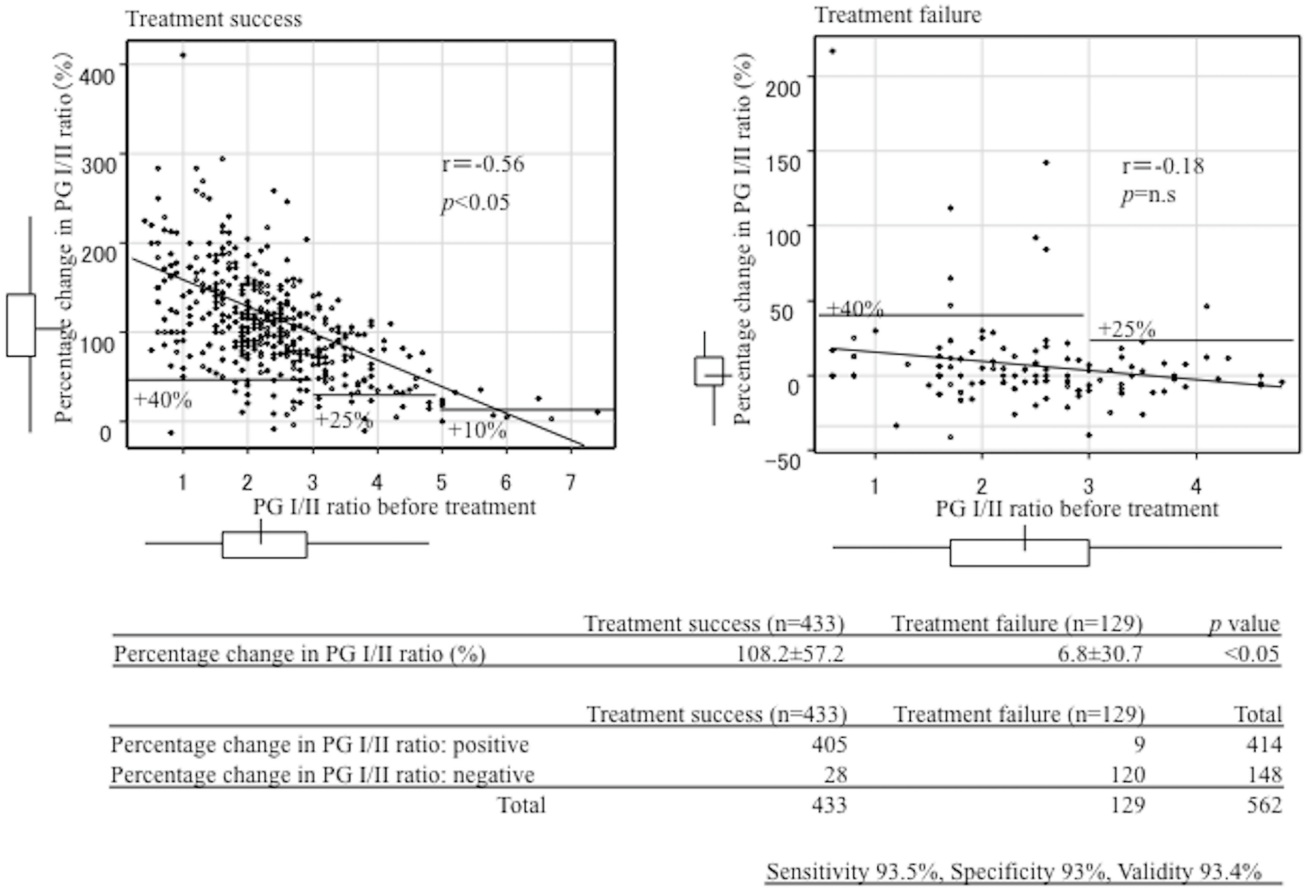
Correlation between the percentage change in the PG I/II ratio and the PG I/II ratio before treatment.

The increasing percentage in the serum levels of PG I/II ratios after treatment compared with the values before treatment clearly distinguished success from failure of eradication (108.2±57.2 vs. 6.8±30.7, *p*<0.05). Using the above cut-off values, the sensitivity, specificity and validity for determination of *H*. *pylori* were 93.1, 93.8 and 93.2%, respectively.

## Discussion

In this study, the percentage changes in serum PG I/II ratios showed high concordance with the outcome of eradication therapy for *H*. *pylori*. In Japan, diagnosis methods of *H*. *pylori* infection are RUT, microscopic examination, culturing method, UBT, anti-*H*. *pylori* antibody measurement, *H*. *pylori* stool antigen test. Among them, the UBT [18] [19] and the *H*. *pylori* stool antigen test [20] are useful for the diagnosis of *H*. *pylori* infection after eradication treatment, and are usually performed 4 weeks after completing the treatment course. The UBT shows both high sensitivity and specificity for determining the success of *H*. *pylori* eradication (sensitivity 95%, specificity 95%) [19]. However, when the measured value is 2.5–5.0‰, there is a possibility of obtaining a false positive result and it is therefore necessary to reexamine using the UBT or another test method. As unlike the serum PG methods which only requires blood sampling, it is necessary to newly space for examination, and to secure new staff. The *H*. *pylori* stool antigen test, which is both noninvasive and simple, also shows high reliability (sensitivity 95%, specificity 97%) even for the diagnosis of infection before *H*. *pylori* eradication treatment [20]. However, collection and processing of stool samples are complicated. In this study, the percentage change in the PG I/II ratio showed sensitivity of 93% and specificity of 92%, which is equivalent to these methods. Therefore, it might be convenient and cost effective compared to above traditional methods.

Plasma PG levels are expected to decrease and remain low following successful eradication. Whereas, PG should remain stable or return to baseline after an initial drop if therapy is unsuccessful [21]. Furthermore, it has been suggested that PG II could be an even better marker for this difference than PG I [22] [23]. This was confirmed by a study by Kawai and colleagues who showed that similar PG I/II levels were found 2 months after eradication and that these levels were comparable 12 and 24 months after treatment [24]. In the follow-up test, the PG I/II ratio showed an increase. Successful removal of *H*. *pylori* results in a decrease in serum PG I and PG II, however, the PG I/II ratio increases as a result of the PG II decrease more than that of PG I. A more rapid decrease in PG II than PGI 1 month following eradication has also been reported by Ohkusa and colleagues [25]. PG I/II is not only a widely accepted marker for atrophy, but also the increase in the ratio is considered to be an indicator of *H*. *pylori* eradication success [26].

There are several reports of using PG for the evaluation of treatment outcomes in patients with *H*. *pylori* infection. PG II decreases of over 10, 15, 25 and 30% or PG I/II ratio increases of over 25, 30 and 68% were reported as single cut-off values. Whereas, both PG II decreases of over 40% and PG I/II ratio increases of over 140% were reported as multiple cut-off values, except for certain criteria, in this study. Previous reports have reported the higher sensitivity and specificity of multiple cut-off values compared with single cut-off values (single cut-off value: sensitivity 61.9–95.7%, specificity 61–97.2%, accuracy 61–95.4%; multiple cut-off values: sensitivity 97.1–100%, specificity 89.8–97.9%, accuracy 90.3–96.2%) [27–33]. This finding was verified in our study. When multiple cut-off values are determined by assessing *H*. *pylori* eradication based on serum PG values, it is necessary to change the cut-off value according to the PG I/II ratio before treatment. Furuta and colleagues reported that the average rates of change in the case of pre-treatment with a PG I/II ratio of below 3.0, above 3.0 but below 5.0, and above 5.0, in 292 patients in which eradication of *H*. *pylori* was successful were 120.7, 75.0 and 37.5%, respectively [17]. Therefore, the change in the serum PG I/II ratio was significantly different depends on the pretreatment value of the serum PG I/II, especially when the pretreatment serum PG I/II ratio was low. This was because the group that had low serum PG I/II ratio before treatment had high gastritis activity scores. In this group with more severe gastritis, the change in serum PG I/II ratio before and after *H*. *pylori* eradication might be greater. Therefore, when we examine the judgment of *H*. *pylori* eradication according to the rate of change of the serum PG I/II, the lower the serum PG I/II ratio before treatment, we should set the higher the cutoff value. On the other hand, when the PG I/II ratio before treatment is high, we should set the lower cut off value. These might be necessary to achieve high accuracy.

The present study has some limitations. It was a retrospective study with a small study cohort. In this study, 37/562 (6.6%) patients were not successfully assessed using the percentage change of PG I/II (Insufficient increasing in the pepsinogen I/II ratio: 31/37 (83.7%), false positive of UBT: 2/37(5.4%), false negative of UBT: 4/37 (10.8%)). Therefore we will validate such cases and aim at higher sensitivity and specificity by setting an appropriate cut-off level with more patients in the near future.

In conclusion, our findings suggested that the percentage changes in serum PG I/II ratios are useful as evaluation criteria for the success of eradication therapy for *H*. *pylori*.

## Supporting information

S1 Fig The best cut off value of percentage change of PG I/IIratio for all patients. (JPG)

S2 Fig The best cut off value of percentage change of PG I/IIratio for the patients with atrophic gastritis. (JPG)

S3 Fig The best cut off value of percentage change of PG I/IIratio for the patients without atrophic gastritis. (JPG)

S4 Fig The best cut off value of PG II change in percentage for all patients. (JPG)
