# Supplementary figures and images for: A significant increase in the pepsinogen I/II ratio is a reliable biomarker for successful Helicobacter pylori eradication
Source: PLoS One. 2017 Aug 30;12(8):e0183980. doi: 10.1371/journal.pone.0183980 (PMC5576670; doi:10.1371/journal.pone.0183980)

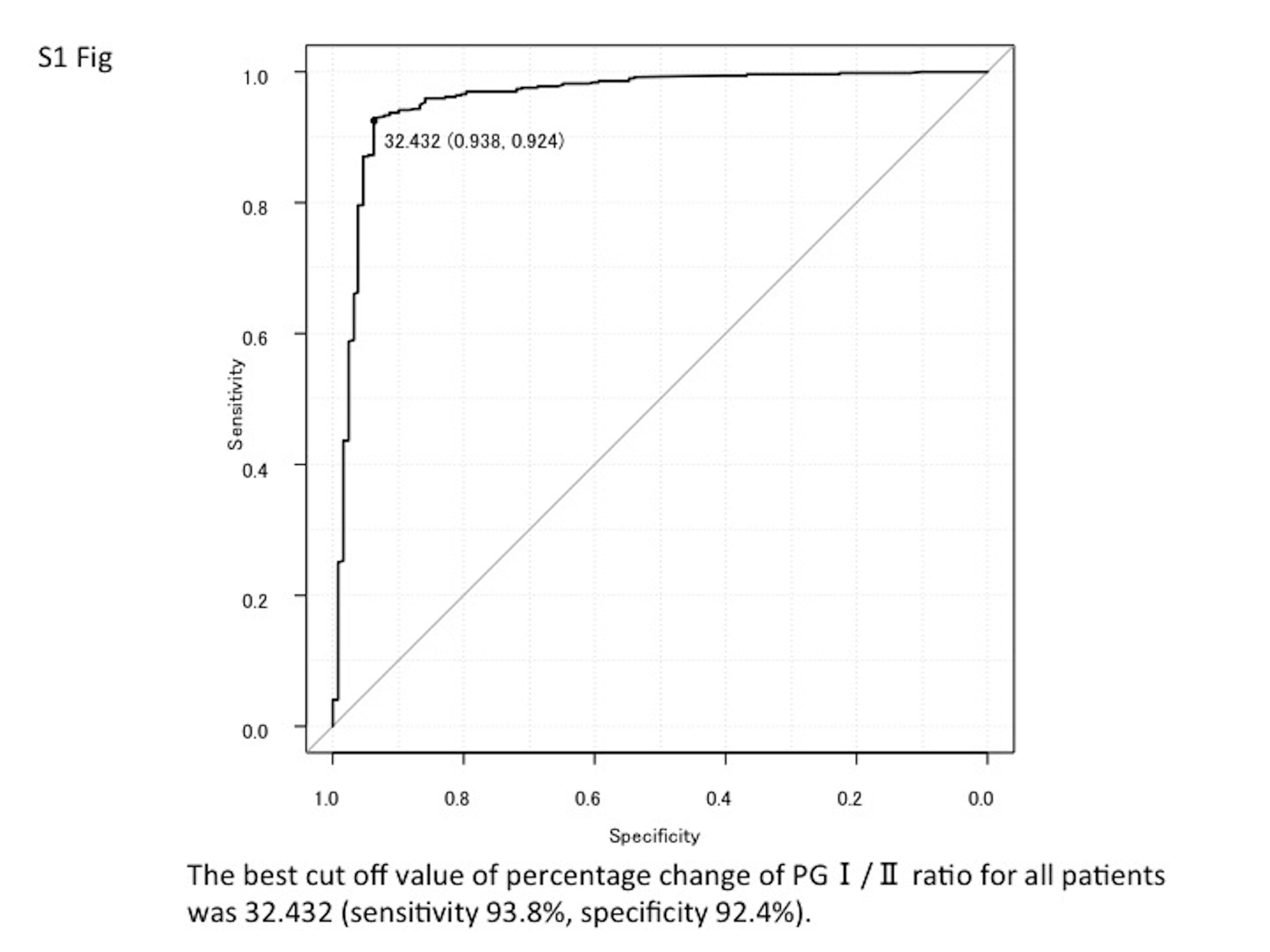

Supplement: S1 Fig — (JPG) [file pone.0183980.s001.jpg]

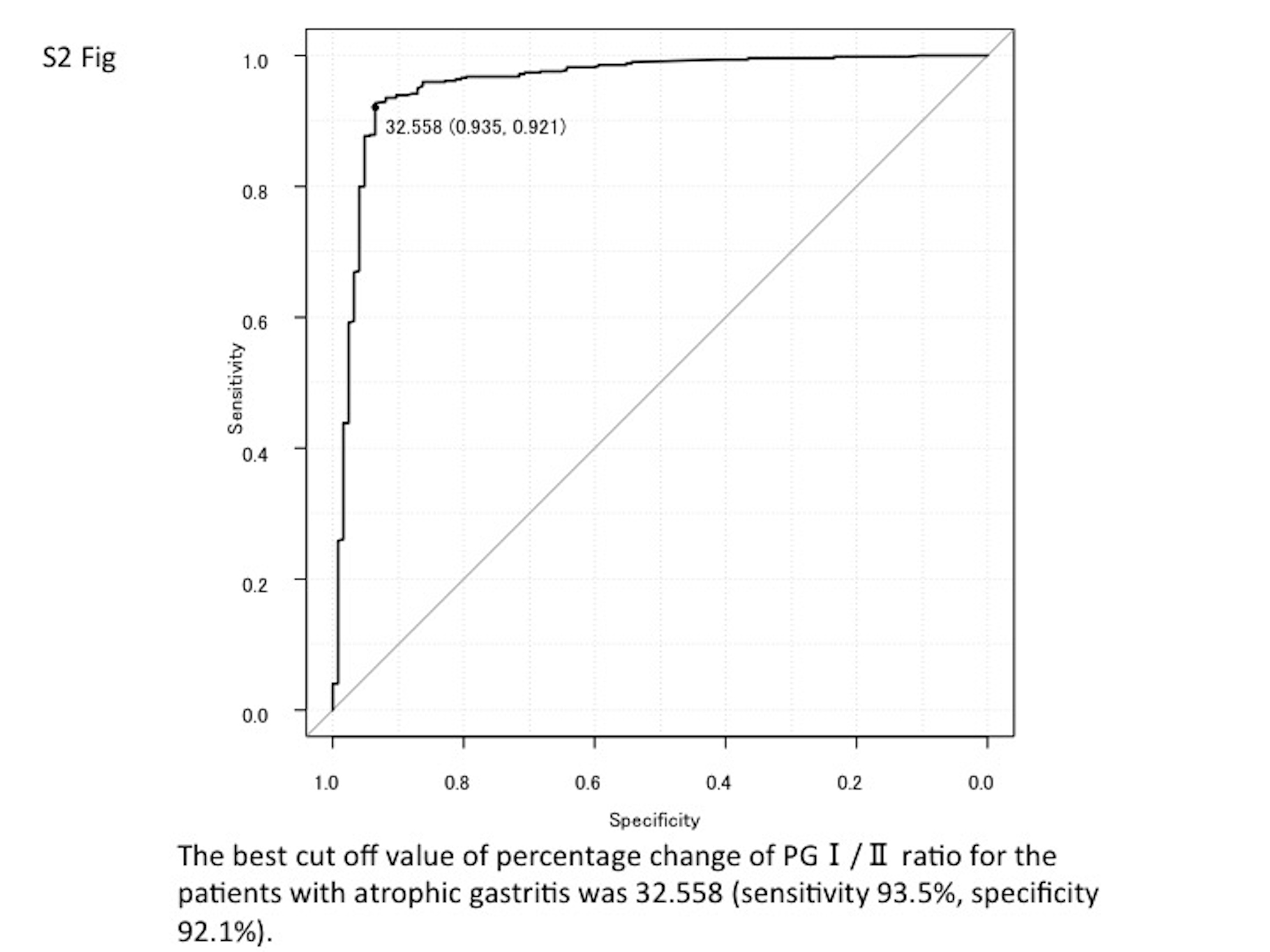

Supplement: S2 Fig — (JPG) [file pone.0183980.s002.jpg]

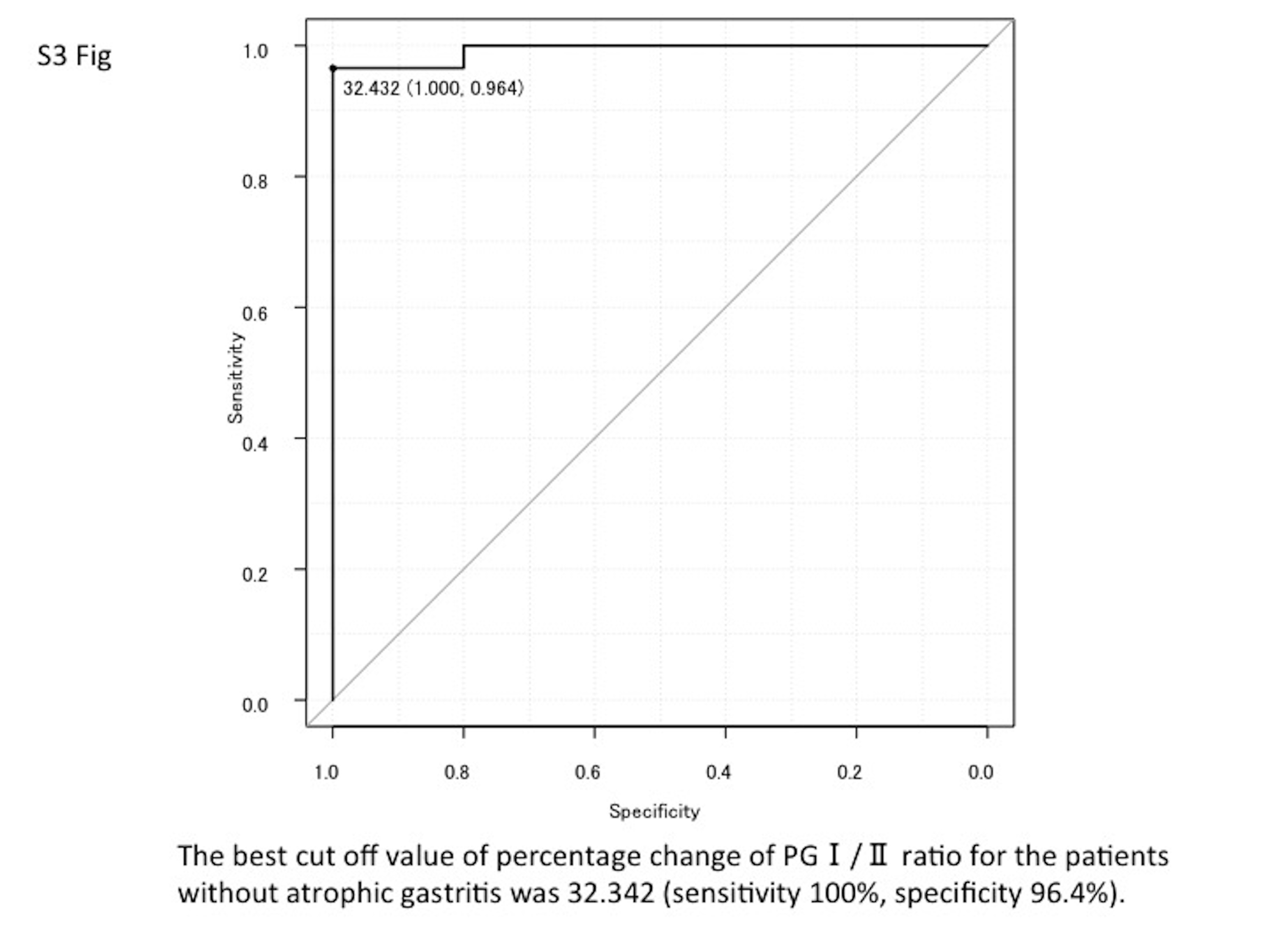

Supplement: S3 Fig — (JPG) [file pone.0183980.s003.jpg]

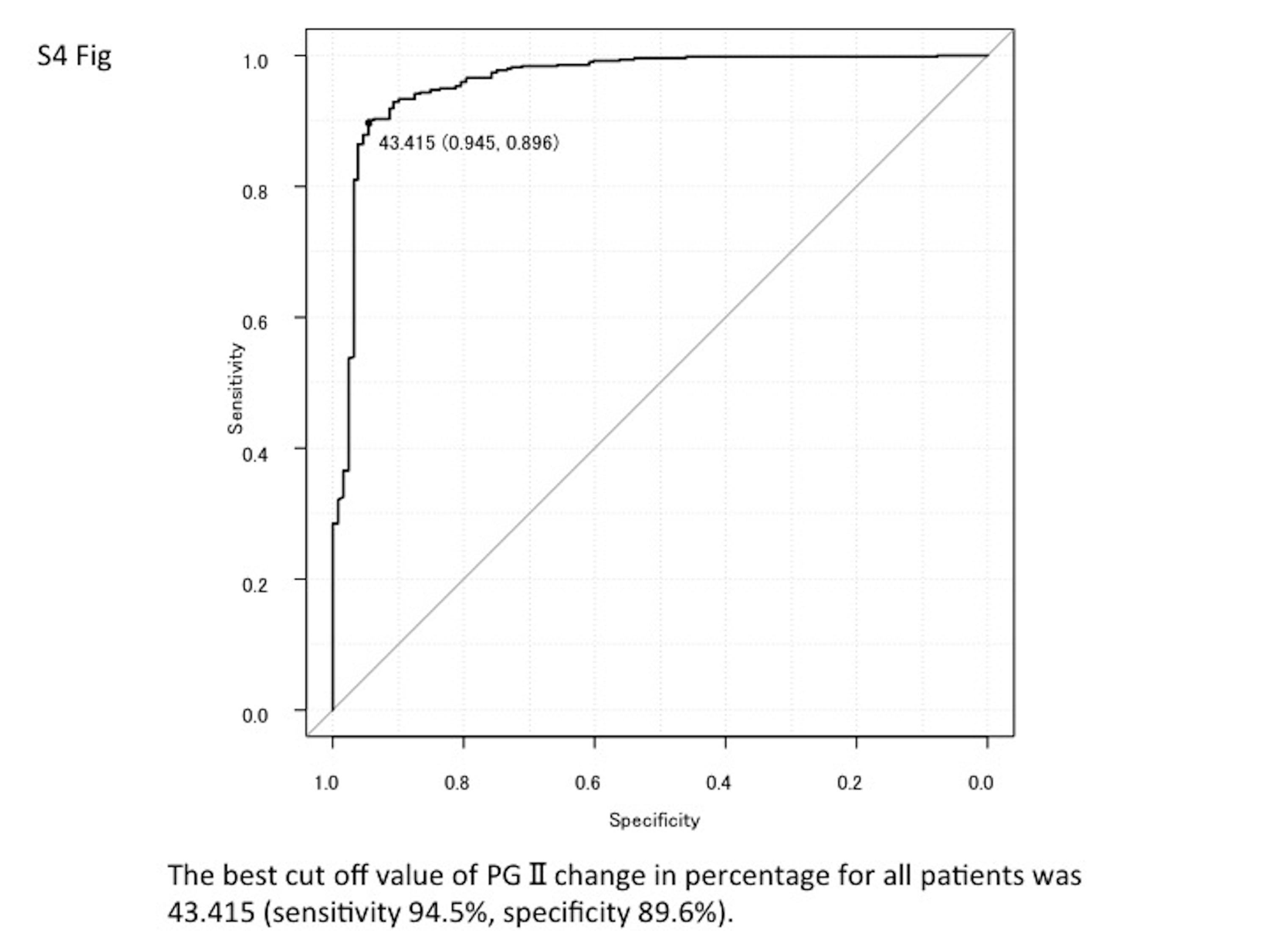

Supplement: S4 Fig — (JPG) [file pone.0183980.s004.jpg]
